# Beyond the Classroom: Investigating the Relationship between Psychomotor Development and Academic Achievement in 4–12-Year-Olds

**DOI:** 10.3390/children11080973

**Published:** 2024-08-12

**Authors:** Nídia Amorim, Adilson Marques, Sofia Santos

**Affiliations:** 1 CEE—Center for Studies in Education, Faculty of Human Motricity, University of Lisbon, 1499-002 Lisboa, Portugal; nidiaamorim@fmh.ulisboa.pt; 2 Interdisciplinary Center for the Study of Human Performance (CIPER), Faculty of Human Motricity, University of Lisbon, 1499-002 Lisboa, Portugal; 3 Education and Training Research and Development Unit—UIDEF, Education Institute Faculty of Human Motricity, University of Lisbon, 1499-002 Lisboa, Portugal; sofiasantos@fmh.ulisboa.pt

**Keywords:** psychomotricity, learning skills, child-centered planning, developmental disorders, pre-school, school, students, kindergarten, childhood

## Abstract

Background/Objectives: The relevance of psychomotor skills in children’s growth is being increasingly recognized. The transversal role of psychomotor skills in learning performance is described through a link between cognitive and motor functioning, promoting socio-affective–expressive competencies, but there is a scarcity of evidence from the field. A two-fold goal was defined: to investigate the relationship between psychomotor functions and academic performance and to examine the factors affecting children’s academic performance. Methods: The Portuguese versions of the Neuropsychomotor Functions Assessment Battery for Children (NPmot.pt), Preschool Diagnostic Tasks (PRE), and School Learning Skills Battery (SLSB) were applied to 350 children (85.72 ± 24.23 months) with and without disabilities attending mainstream schools. Results: Pearson correlations and regression analyses were used. NPmot.pt domains showed moderate to strong correlations with PRE domains (0.30 < r < 0.82) and weak ones with SLSB domains (r < 0.30). Psychomotor development is a stronger predictor (*p* < 0.001) of pre-academic performance outcomes: (βTonus = 0.67, βGross Motor Skills = 1.04, βSpatial Orientation = −1.44, βRhythm = −1.59 and βAuditory Attention = 3.68) than of academic performance above 7 years old (*p* > 0.05). Conclusions: Results strengthen the importance of psychomotor skills development from an early age, also at school, with implications for an early psychomotor assessment and intervention for children with and without disabilities. Tailor-fit interventions, including strategies to improve psychomotor skills, should be promoted during the school process of all children for a successful learning process.

## 1. Introduction

Childhood is a developmentally rich stage in an individual’s life: after achieving bipedal skills and some language, children begin to explore their surroundings more autonomously, acquiring and consolidating new skills from a psychomotor, linguistic, social and cognitive point of view [1], contributing to healthy growth [2,3]. In this period of life, between 3 and 11 years old, learning takes place mainly through movement in the initial phase (from 3 to 6 years old), and then moves on to a phase of greater specialization of gesture associated with greater expression of verbal skills and a progressively more abstract scope [1,3]. In both periods, psychomotor skills emerge as crucial elements of human development, since by integrating cognitive, emotional and symbolic functions, the body is seen as a fundamental tool for expressing individuality and interacting with the world. Movement, in this context, goes beyond mere physical action and becomes a language through which the individual communicates, learns and adapts to their environment [3,4,5,6,7]. 

Psychomotor skills, as an expression of psychomotor development, help children to mentally organize the outside world through body movements. The early development of psychomotor skills supports children in acquiring mastery over their bodies through balance and coordinated movements, preparing them for the challenges of everyday life [1,2]. Furthermore, from a cognitive point of view, the development of psychomotor skills improves attention span, concentration and memory [8] as well as promoting creativity [5], and from the social point of view, facilitates more frequent interactions with other people, allowing children to engage with the wider social environment [1,5]. Defining and analyzing a child’s psychomotor profile throughout their development is extremely important, both for the earliest possible diagnosis and for drawing up valid assessment processes and planning and monitoring person-centered interventions [9].

Children tend to spend most of their day in school, engaged in reading, writing and mathematics activities [10], demanding psychomotor skills that act as the basis for early literacy and number knowledge and may influence onward development and procedural knowledge [10,11,12,13]. These skills are fundamental to overall development and a good starting point to generate learning in other knowledge dimensions [5,10]. The individualized teaching–learning path of each pupil should be structured based on the early identification of a psychomotor profile during childhood [14]. Cameron et al. (2016) [15] analyzed the motor coordination, executive functions and visuospatial skills of pre-school children, exploring the relationship between psychomotor skills and literacy and numeracy development, and concluded on the need to implement strategies and methodologies that promote psychomotor development as a complementary and supportive means of robust academic learning. Learning through the motor act allows the brain (prefrontal cortex) to learn the gesture and associate the appropriate response to the situation with the respective processing areas. This process will allow that learning to be automatized, leaving the body free for new challenges, with previous acquisitions supporting sustained learning [15]. Gross and fine motor skills are related to academic success, namely, abilities of literacy (decoding letters and words), writing, numeracy (solving quantitative problems), and socialization [15,16,17,18,19,20]. Studies on fine motor skills and literacy have emphasized visuomotor integration [18,20]. When accounting for executive function, visuomotor integration measures are found to uniquely predict early print knowledge in preschoolers [21], as well as improvements in letter–word identification, print knowledge, and phonological awareness in kindergarteners [22].

The aforementioned correlation can be elucidated by the Cattell–Horn–Carroll theory of cognitive abilities in which psychomotor and academic domains influence each other. Psychomotor skills contribute to the acquisition and consolidation of academic skills such as mathematical knowledge, reading and writing through spatial and temporal orientation, visuomotor and perceptual skills [23]. The human psychomotor system’s conceptual model [24,25,26] is based on a multidimensional and hierarchical system, where the several psychomotor factors correlate with each other according to their respective neurological maturation and degree of specialization. In this way, it can be seen that tonus and gross motor skills (including balance) appear as the first references that children demonstrate in their interaction and exploration of the environment [24].

Tonus, an adaptive response to gravity, is responsible for all human motricity and stems from a cephalo-caudal and proximal-distal maturational process [24]; it is subdivided into four types [24,25,26]: *rest* (posture and quality of the movement) [24,25,26], *action*, *support* and *attitude*, allowing the individual to adjust appropriately to the situation. These all demonstrate the functional organization of the brain and cerebral hemispheres, as well as reflect the child’s affective and emotional regulation [25]. The presence of tonic alterations (hyper- and hypotonia, and/or synkinesis) interferes with the ability to learn, since the body is not available for the various cognitive–academic demands [24]. Fonseca [24] added the impact on language acquisition and the development of fine motor skills.

Balance is crucial for the acquisition of bipedal posture and locomotion [24]. Tone and balance issues affect posture, gait, processing of visual information and movement coordination, which compromise more specialized skills, such as manipulating objects with impacts on academic learning [27,28] such as writing [24,26] or math [28]. Both static and dynamic balance show moderate and significant correlations with language, spatial orientation, and math [28], which may be lower in the presence of other motor skills, such as speed, agility and hand–eye coordination [29]. 

Laterality, the ability to differentiate and recognize the right and left sides of the body, tends to mature by the age of 5–6 years, being fully consolidated around 10 years of age [30,31], even if a hand preference is noticeable at around 3 years [21]. Difficulties in these skills jeopardize the production and performance of motor gestures and their transfer into the school context, leading to commitments in academic learning [4,5,12,32,33]. Difficulties in laterality are correlated with difficulties in learning to write, particularly with regard to spatial orientation [34]. With these acquisitions naturally comes greater body awareness (from the age of 3–4), since manual preference will give greater confidence and autonomy [35], through the dissociation of movements and coordination between time and space [36,37].

The ability to recognize body segments, the kinaesthetic sense [24,30], as well as their location in space (proprioception), allows children to acquire spatial concepts (e.g., down and left) and temporal concepts (e.g., order and succession), seeing themselves as a reference point for their environment’s organization [37]. This will make it easier to improve imitation processes through the maturation of mental representations and the acquisition of the symbolic [11,32], thus accessing increasingly abstract concepts such as calculation-related skills [38]. The body can be used as a mediator in learning the alphabet and letters [35].

Hand–eye coordination and manual praxis involve the sequencing of movements essential for motor planning, linking visual perception with hand/foot motor skills, and establishing somatognosia [24,25,30]. Bimanual coordination requires a manual preference to support actions and becomes more specialized from second childhood, closely linked with visual discrimination, spatial skills, and the understanding of colors, shapes, and sizes [24,25,35,39,40]. These skills impact graphomotor and lexical processing, as well as vocabulary and language acquisition during the preschool period [41,42]. Bilateral coordination activities, such as jumping, are crucial for reading, writing, and mathematical reasoning in school-age children [27,28]. Gross motor skills, particularly object manipulation, enhance academic performance in math and reading, with strong correlations between fine motor skills, self-regulation, literacy, writing, and problem-solving skills [18,28,43,44,45,46,47]. Visuomotor coordination, cognitive flexibility, and inhibitory control are vital for understanding syllables and the alphabetic principle, as well as for the motor control needed in writing and reading tasks [35,48,49]. The visuomotor system enables the detection and discrimination of stimuli, identifying details such as shapes and boundaries [24,50]. Fine motor skills are closely related to lexical processing, reading, writing, and mathematical reasoning due to the progression from symmetrical to homolateral and then combined movements, supporting writing development [17,47,49,51,52]. Visuomotor coordination is linked to visual perception and mental representation during visual integration, necessary for activities such as reaching, holding, reading, drawing, writing, and mathematical reasoning [48,53].

Spatial orientation, rhythm, and auditory attention require the integration of spatial information (e.g., distance, size, notion of depth, and perspective) and rhythmic understanding [24,25], which begin to develop from the age of two and become more evident in the school context [54,55], and are strongly correlated with academic performance [56,57]: spatial orientation is associated with mathematical reasoning and concepts [58], and temporal orientation is related to various linguistic constructs [25] essential even before formal schooling [59]. Rhythm, pre-literacy, and auditory attention seem to be interlinked at preschool age [59]. Verbal acquisition includes both expression (production and speech) and comprehension (receptive language, understanding, and listening), and is based on phonological, lexical, and grammatical characteristics, influenced by the quantity and quality of literacy stimulation and materials diversity [60]. 

Quantitative concepts or early mathematical skills are strong predictors of future educational success. Learning to count sequentially involves retaining previous information, updating it with new information, and inhibiting interference from other numbers [61]. There is an outstanding relationship in the logical–mathematical domain between 3 and 8 years [12]. The consolidation of short-term memory through activities such as walking, jumping, and maintaining balance seems to contribute, albeit indirectly, to mathematical success [62] and to verbal and non-verbal comprehension, memory, receptive and expressive language, and communication skills [60,63].

Children naturally and frequently use their fingers to assist with arithmetic activities, which allows for the representation of quantities in an analog manner before symbolic representations’ development, and enables calculations [64]. Using an ordered and stable sequence of finger movements when counting helps children memorize counted elements by relying on the one-to-one correspondence between the fingers raised and the objects counted [65]. This is crucial for understanding cardinality, i.e., the ability to accurately determine the size of a collection. Children who most often use their fingers to calculate in preschool are typically the best at arithmetic tasks [65]. Dexterity is significantly correlated with additive computation skills but not with vocabulary skills [66]. The training of fine motor skills enhances numeracy performance [56], becoming explicit in children around 5 years old [66]. 

Finally, auditory memory, the ability to store phonological information for short periods, allows for temporary manipulation—this manifests itself when children are asked to repeat longer and longer sequences of numbers and words, with or without meaning [57]. The phonological cycle (verbal–phonological information), visual sketching (visual and phonological information) and central executive systems (attention regulation) are essential processes that begin in early childhood and develop along with the increase in vocabulary between 12 and 36 months, with this relationship continuing to grow during the pre-school phase [57]. Better psychomotor development reduces cognitive and school learning difficulties, including auditory attention [67]. The better the ability to capture and retain what has been heard, the less likely it is that learning difficulties and impacts on other areas of children’s development will arise [30]. 

The education system in Portugal consists of four stages (pre-school, primary, secondary and higher education). Formal education is universal, free and mandatory between 6 and 18 years old [68]. Early childhood education has gained increasing interest worldwide, due to its key role in developmental and educational success, with children attending the pre-school system (not compulsory) exhibiting more academic advantages [11]. Pre-school education the first stage of the lifelong education process, aims to promote the child’s development (3–6 years old) through lifelong learning [14] and is still optional in Portugal, although a strong commitment and desire exists to make it universal from the age of four [14,68]. The main goals of pre-school education are to create opportunities and experiences at the personal, social, recreational, moral, emotional, civic and psychomotor levels, promoting communication and language, critical thinking, and curiosity, key elements in any learning process [14]. It also enables the screening of deviations in neurotypical development, thus enabling appropriate early intervention [14]. Pre-school education should adopt a multidimensional approach, integrating psychomotor, cognitive, and affective aspects for comprehensive academic learning, including areas such as verbal and quantitative concepts, auditory memory, shape constancy, positions in space, spatial orientation, visuomotor coordination, and figure-ground perception [69]. 

Primary education is characterized as a period of learning and consolidation of basic and general skills, guaranteeing an overall education common to all citizens. This formal education process should enable all children to learn and develop their interests and skills, as well as their reasoning, memory and critical thinking skills, creativity, moral sense, and aesthetic sensibility, towards functionality, social participation and citizenship within inclusive environments that are conducive to the development of all children’s academic and educational success [70]. Children exposed to high-quality learning processes at an early age are more likely to experience academic success [12,63,71].

The specificity and combination of all these factors makes it crucial to identify the psychomotor profile and correlate it with academic performance, emphasizing the need for understanding the challenges faced by all children (with or without disabilities) for tailor-fit interventions. A holistic approach to psychomotor interventions within the educational system may empower academic performance. Although evidence points to an expected and significant correlation between psychomotor skills and academic achievement, with psychomotor ability a critical issue for the identification of children at risk of educational underachievement, this is a field still under-explored with a need to be cautious with this interpretation, given the small sample size and lack of replication studies [51]. The trend is still towards conventional academic approaches. Due to the widespread attention to scholastic success and the need for targeted interventions beyond conventional approaches, and trying to contribute to a more comprehensive understanding of the impact of psychomotor skills on successful learning processes, our goal was to analyze the correlations between psychomotor skills and academic performance at both pre-school and school ages.

We began by analyzing the correlation between variables from three instruments designed to assess each of these areas, assuming that there would be moderate to strong correlations between them. Further, it was investigated how the variables of sex, year of schooling, and the presence of specific learning difficulties affect this relationship. Literature indicates that the maturational curve stabilizes by the age of 8 years. Better results are expected in the pre-school age groups. This study will allow for understanding of the importance of the psychomotor profile in the context of educational achievements, outlining priorities for pedagogical intervention with children in these age groups, since psychomotor stimulation promotes the development of fundamental skills for school learning [72,73,74].

## 2. Materials and Methods

### 2.1. Participants

This study involved a convenience sample of 350 children, aged between 46 and 154 months (85.72 ± 24.23), of which 170 were female and 180 were male, attending school since kindergarten (n = 143, 40.9%) up to the eighth year (n = 207, 50.1%). Most students had a typical development trajectory, and only 17 had specific learning needs (SLN). The majority of participants studied at private schools (n = 219; 62.6%) and the remainder at public schools (37.4%). Students with SLN were mainly boys attending public schools in pre-school education (n = 5, 4–6 years old) or primary education (n = 12), with the majority enrolled in private schools (n = 9). The inclusion criteria were: ages between 4 and 12 years, enrolled and attending school (home schooling was not considered), without any clinical conditions such as severe motor and/or sensory issues that would prevent the instruments’ application. To calculate the sample sizes needed to detect a relevant simple correlation with specified significance level and power, the following criteria were used: α (_probability of type I error_) = 0.05, ß (_probability of type II error or power of the test_) = 0.10 and r = −30. For each subgroup the number was 158, which in total demanded a (minimal) total sample of 316.

### 2.2. Instruments

The Portuguese version of the *Neuropsychomotor Functions Assessment Battery for Children* (*NPmot.pt*) assessment tool, designed to evaluate neuropsychomotor functions, underwent to a rigorous translation and adaptation process to the Portuguese language and culture [75]. The NPmot.pt comprises 52 items distributed into 9 psychomotor domains: tonus, gross motor kills, laterality, manual praxis, tactile gnosis, hand–eye coordination, spatial orientation, rhythm and auditory attention. While these domains can be assessed individually to address specific functions, a comprehensive evaluation across all domains provides a more comprehensive understanding of the overall neuropsychomotor development profile, aiding in the identification and analysis of differential diagnoses [75]. Each item is scored from a quantitative perspective reflecting the child’s performance according to age, ranging from 0 (no performance) to 2 or 5 (indicating optimal performance, depending on the item), complemented by a qualitative assessment of the gesture’s components (e.g., quality, precision, and speed) allowing for detection any deviations from what is expected from a harmonious movement. The assessment is norm-referenced, with each score analyzed through a mean–standard deviation relation based on normative data. This approach enables the establishment of a comprehensive neuropsychomotor profile allowing for the identification of strengths and weaknesses. Data will help to design and implement adjusted and tailor-fit interventions.

The content validity of the NPmot.pt version was obtained by expert agreement, with nine experts rating each item according to its relevance, clarity, simplicity, and ambiguity. All content validity indexes (IVC > 0.78) pointed to the items’/indicators’ representativeness, which was corroborated by the strong agreement among experts (>0.42) and Cohen’s kappa scores (0.02 < k < 0.95). Reliability was analyzed through internal consistency (α > 0.45) and temporal stability (test–retest technique), of which scores ranged from 0.45 to 0.99. Correlations between domains and totals ranged from moderate to strong relationships (0.31 > r < 0.92) and an exploratory factorial analysis identified the multidimensionality of the construct, presenting an eight-factor solution that explains 88.5% of the total variance. The NPmot.pt was able to discriminate children with and without developmental disorders (*p* < 0.05). For more details please see [75].

Two different instruments were used to assess academic skills in a school setting.

The *Portuguese Preschool Diagnostic Tasks* (PRE) [76] is a battery of tests designed to assess children’s maturity (aged 4–6 years old) before entering into compulsory schooling within the following areas: Verbal, Quantitative Concepts, Auditory Memory, Visual Perception–Shape Constancy, Spatial Orientation, Positions in Space, Visuomotor Coordination and Visual Perception–Figure–Background. Scoring is conducted by awarding one point for each correct answer and then divided into two categories: either the number of correct answers is added up directly, or the difference between correct and incorrect answers is calculated. Reliability was confirmed through internal consistency with acceptable scores (0.32 < α < 0.95 ) and criterion validity was analyzed through correlations between total score and teacher assessment (r > 0.54). Intercorrelations between areas indicated mostly moderate to strong relationships, with the strongest correlations between verbal comprehension and quantitative concepts, and between quantitative concepts and spatial orientation.

The Portuguese School Learning Skills Battery (SLSB) [77], for children aged 7 to 12 attending school (first and second cycles of basic education), aims to assess basic skills needed for academic learning in the following areas: verbal comprehension (vocabulary), numerical knowledge (quantitative concepts and number use), and perceptual–spatial aptitude (spatial relationships, shape constancy, and spatial orientation), enabling the early detection of potential reading, writing, and mathematics difficulties. Each item is rated according to the accuracy/quality of pupil performance, ranging from 0 (no/wrong answer) to 2 (excellent performance). The split-half method, used for reliability analyses, presented acceptable scores (r > 0.40), and criterion-related validity showed a trend of moderate (0.39 < r < 0.61) correlations between teachers’ perception of children’s performance vs. the respective results.

### 2.3. Procedures

#### Administration and Statistical Analysis

The institutional research ethics committee of the Portuguese Psychomotor Association approved the study procedures (reference 2021/1), which was conducted in accordance with the Declaration of Helsinki and with all ethical requisites fulfilled. Then, contacts were established with several schools’ principals to seek permission to administer the instruments in the scholastic community. An informed consent document was given outlining the research’s goals, procedures and ethical considerations, assuring confidentiality, privacy and anonymity of data. When authorization was granted, a similar process was conducted with parents/legal guardians who provided written authorization. After the collection of these signed documents, and after all children agreed to participate, the instruments were applied within the school context. The NPmot.pt was applied at the school’s gym and the other instruments in the classroom, in previously agreed upon spaces and moments, trying to interfere the least possible with each child’s academic and extracurricular activities. 

All instruments were applied according to their own protocols. The NPmot.pt took approximately 90 min/child) and the academic instruments around 60 min/class. 

Statistical analyses were carried out using Statistical Package of Social Sciences (SPSS) software, version 28. The parametric test of Pearson correlations was applied in order to analyze potential relations between all instruments’ domains and total scores. Potential differences were analyzed based on sex, scholar level/age and diagnosis. Multiple regression analysis was performed to evaluate the relationship between the three instruments applied. The significance level was set at *p* < 0.05.

## 3. Results

The correlations between psychomotor functions and preschool and school capabilities were analyzed and are presented in Table 1. Subsequently, analysis was conducted based on sex (Table 2) [78], grade level (Table 3), and the presence of special needs (Table 4) [79], with a significance level set at 0.05. These variables were chosen in accordance with the studies carried out by Peyre and his team and Blank et al., with the aim of understanding the present correlations. Result interpretation followed these criteria [80]: weak if r < 0.40, moderate if 0.40 ≤ r < 0.70, strong if 0.70 ≤ r < 0.89 or very strong if r > 0.90.

The correlations between psychomotor and pre-academic skills varied from weak (r = 0.20) to strong (r = 0.84), with the most significant correlations (** *p* < 0.01) between *tonus*, *laterality*, *manual praxis*, *gross motor skills* (NPmot.pt) and the *verbal*, *quantitative concepts*, *visual perception*, *visual–motor coordination* and *total* sub-scales (PRE). It should be noted that the *verbal* and *quantitative concepts* sub-scales were significantly correlated with moderate to strong correlations with all the psychomotor domains, with the exception of *spatial orientation* (NPmot.pt) with *quantitative concepts* (PRE). Additionally, with regard to the PRE scale, the areas of *spatial orientation* and *auditory memory* were those with the highest number of statistically insignificant correlations. *Gross motor skills* is the psychomotor domain that showed significant correlations with all PRE domains. The total for both instruments seemed to correlate moderately and significantly. 

As can be seen in the table, the correlations between SLSB and NPmot.pt were almost non-existent, and those that are considered statistically significant were weak (0.14 < r < 0.24). The psychomotor domains of *laterality* and *rhythm* correlated with the academic dimensions in general, which is reflected in the total of both scales and between the totals and the domains of the two instruments (r = 0.17). The psychomotor domains of *tonus*, *gross motor skills*, *tactile gnosis* and *auditory attention* did not show statistically significant correlations with any of the academic domains.

Analyzing by sex (Table 2), the correlations between PRE and NPmot.pt show the same variation between weak (r < 0.15) and strong (r < 0.90) correlations, despite a greater tendency towards moderate correlations. In both sexes, the most significant correlations (*p* < 0.01) were between *gross motor skills* and all PRE domains except for *spatial orientation* and *auditory memory*; the same was true for the psychomotor domain of *manual praxis*.

The total NPmot.pt seemed to correlate with all the PRE domains (*p* < 0.01), showing moderate to strong and significant values (0.48 < r < 0.83) in most of them, with the exception of *auditory memory* and *spatial orientation*, where it showed significant but weak correlations (0.16 < r 0.25).

A trend of weak to strong correlations, highest in the last year of pre-school, is presented when analyzing by year of schooling (Table 3). Very weak or non-existent correlations were found from the third year of schooling onwards, in the first grade. The correlations between the two instruments’ totals showed weak scores for pre-school education (r = 0.31) and non-significant ones in the remaining years of schooling. Moderate correlations were found in the last two years of pre-school education, with moderate to very strong values for the *verbal subscale* with various psychomotor domains. The spatial skills presented the lowest values (e.g., r = 0.27, visual perception–figure-bottom; r = 0.27, visuomotor coordination; r = 0.31, auditory memory; r = 0.29, shape constancy). *Gross motor skills and rhythm* are the psychomotor domains with the most consistent correlations with the preschool domains.

Almost all correlations between psychomotor domains and SLSB instrument tasks are non-existent, with exceptions being between: *verbal comprehension* and *laterality* (r = 0.78), *spatial orientation* and *manual praxis* (r = 0.67), *spatial orientation* and *eye-hand coordination* (r = 0.89), *spatial orientation* and *auditory attention* (r = 0.70), and *total SLSB* and *auditory attention* (r = 0.75).

When analyzing the correlations between all instruments’ domains and the existence or not of SLN on the part of the children (Table 4), the most significant correlations were found in the school-age population (from the first year of the first cycle), with the most significant being *manual praxis* (0.18 < r < 0.91), *gross motor skills* (0.45 < r < 0.84), *spatial orientation* (0.30 < r < 0.61) and *auditory attention* (0.19 < r < 0.59), as well as between the *totals* of NPmot.pt and SLSB (0.20 < r < 0.83). In addition, between these two scales, the lowest score was associated with *rhythm* (0.28 < r < 0.31), while all others were considered moderate to strong. 

Regarding correlations at pre-school age, there was a notable absence of significant correlations in the group of children with SLN. The lowest correlations were between the PRE’s domains and *laterality* (0.18 < r < 0.30) and *rhythm* (0.26 < r < 0.36). There were no correlations between the *tactile gnosis* domain and PRE domains. However, we can observe some moderate to strong correlations: *manual praxis* (0.18 < r < 0.91), *gross motor skills* (0.45 < r < 0.84), *totals* of the two scales (0.20 < r < 0.83), *spatial orientation* (0.30 < r < 0.61) and *auditory attention* (0.19 < r < 0.59).

Table 5 showed the regression analyses between NPmot.pt and PRE and between NPmot.pt and SLSB.

Psychomotor development explained 58% of the variance in pre-school academic performance but did not seem to predict academic skills with the SLSB scale, explaining only 5.2% of academic skills variability. 

## 4. Discussion

The essential contribution of our study lies in analyzing the correlations between psychomotor profile and academic performance at pre-school and school age (4–12 years). It allows us to reflect on which of the constructs appears to have the greatest influence on predicting the performance of the other, i.e., whether good psychomotor performance predicts good academic performance, and attempts to go beyond (academic) conventional approaches, based mainly on cognitive content massification. A unique contribution of this study is related to the scope (broader perspective of both academic and psychomotor components) and context (Portugal) of the data collected. Our findings support evidence of moderate to strong correlations between psychomotor skills and academic capabilities [13,62,68,72], outlining priorities for pedagogical intervention with children in these age groups [8,23,63,74]. 

Results pointed to a trend towards weak-to-moderate correlations between psychomotor (except *spatial orientation*) and pre-academic skills, with the most significant involving *tonus*, *gross motor skills*, *laterality*, *manual praxis*, *rhythm* (NPmot.pt) and the *verbal* [21,24,25,34,59,66], *quantitative concepts* [15,21,56,61], *visual perception* [15,51], *visual–motor coordination* [20,48,49] and *total* sub-scales (PRE). These findings may be explained by the process of neurological maturation, which promotes high growth and plasticity [1,2,3,7,25], with *tonus* acting as the foundation of learning [24,25]. *Laterality* and *rhythm* are moderately to strongly correlated with academic dimensions in general, especially at earlier ages [30,31], due to their presence in the production of coordinated motor gestures [4,5,12,27,28,32,33] in reading [35], writing [34], math [38] and spatial problem-solving [35,36,37]. The link between rhythm and pre-school literacy corroborates other evidence [59]. *Gross motor skills* and *manual praxis* seem to have a major impact on academic performance [10,18,56,63]. This is easily explained by the need to manipulate objects and use fingers (e.g., to calculate) and by the visuomotor integration required for the initial steps within reading (decoding letters, words), writing and math performance (e.g., number conceptualization, calculation processes, abstract reasoning) [15,16,17,18,19,20,21,22,23,26,35,48,49,51,53,64,65]. These findings tend to support the hypothesized relationship. *Manual praxis* resulting from neuromotor maturation demands imitative behavior or repetition [17,47,49,51] for a sequence of actions towards goals [24,25] and involves digital dexterity, planning, and coordination of asymmetrical hand movements during learning situations. These skills impact graphomotor and lexical process during the pre-school period [41,42]. Psychomotor skills influence early literacy and number knowledge, influencing onward development and procedural knowledge [9,11,12]. The most significant relationships are between psychomotor *laterality* and *rhythm* and *verbal comprehension*, *spatial relationships*, and *numerical skills*, linking reading and mathematical calculation to knowledge of the body’s hemispheres, using the body as an orientation reference point with impact on reading rules (top to bottom, left to right) [9,11,12]. 

It is interesting to note that *spatial orientation* and *auditory memory* were the PRE domains with the most insignificant correlations. *Spatial notions* demand a higher level of abstract reasoning, which is only education-targeted at later ages [24,25,54,55,58,59,73,81,82]. The ability to store information (*auditory memory*) and its consolidation and complementarity with other competences (attention, phonology) tend to be stimulated in the last years of pre-school [30,57,67]. Analyzing the data, it is possible to ascertain a relationship between psychomotor development and academic pre-school success [2,7,13,15,62,63,68]. The strong relationship between pre-academic domains and psychomotor components is understandable, especially between 4–6 years, since body-action and knowledge are the basis of acquiring and consolidating basic psychomotor skills [24,25,26] as well as academic task preparation [26,27,28,29,30,31,35,39,43,44,45,46,47,48,49]. From a cognitive point of view, the development of psychomotor skills improves attention span, concentration, and memory [8], as well as promoting creativity [5] and social development [1,5]. These correlations corroborate previous studies, since psychomotor domains (*gross motor skills*, *eye-hand coordination*, *rhythm* and *manual praxis*) are considered the basis for the development of pre- and academic skills, such as *verbal* skills associated with reading and writing [21,24,25,34,59,66], *quantitative concepts* skills associated with mathematics [15,21,56,61] and *visuomotor* skills associated with coordinated movement in reading, writing and calculation activities [15,20,48,49]. Psychomotor skills support the acquisition of new skills of a more abstract nature, such as academic skills, since they are formed from the movement of exploring the environment. This acquisition of knowledge through the body allows for meaningful and consistent learning, and this symbiotic relationship between psychomotor and academic development has been expressed in various studies [7,13,15,19,62,68,71,72]. 

The psychomotor profile is not correlated with *auditory memory*, *spatial notion* and *visual perception*. This relationship does not corroborate previous studies [5,18,22,33,37,47,50,59], but may be explained by the poor stimulation of these domains in the methodologies implemented in kindergarten and primary school [9].

A major impact of psychomotor scales (*laterality*, *manual praxis* and *rhythm*) is not that visible within formal education, explaining only 5.2% of total variance (vs. 58% in the pre-school period). Moreover, despite presenting significant correlations, the trend is towards weak correlations [30,31,32,33,34,39,41,44,46,51], probably due to the consolidation and stabilization of the psychomotor skills trajectory around 8 years [24,25,72] and the non-specificity of the academic instrument used [76]. Further, the SLSB is characterized as a global screening instrument. 

Based on the need to analyze factors that may impact psychomotor and academic development [4,7,13,68,72], it is interesting to see that sex makes a positive contribution to the relationship between psychomotor and academic performance, tending towards moderate correlation [78] coefficients. Generally speaking, girls perform better in verbal and coordination activities [78], which may explain the higher and stronger correlations found in our study, especially after entering the formal education system. Analysis according to the school year variable reveals positive and significant coefficients again at pre-school age, aligned with the developmental psychomotor trajectory up to the age of 6–7, followed by a period of stabilization and specialization of these skills in the following years [24,25]. This process results from training and greater self-regulation, which frees up the brain for new learning once the primary skills have been consolidated [7,10,24]. The *verbal* subscale is moderately to strongly correlated with most psychomotor domains. The previous trend is seen again: *spatial skills* have lower correlation coefficients in pre-school but stronger correlations in the formal education period, and *gross motor skills* and *rhythm* have the most consistent scores. The findings point out a need for preventive intervention at pre-school age (3–6 years), through exploiting the environment in order to stimulate the acquisition of the fundamental foundations for a healthy academic future in the more formal learning process from the age of six [1,2,3,7,14]. Regarding the existence of specific learning needs, positive and significant correlations were observed between both instruments in children without diagnosis. These data are supported by the fact that the instruments differentiate between the profiles of students with neurotypical and atypical development [75] and that even though the sample is low in expression, it is possible to verify their relationship. However, the reduced sample of children with specific learning needs limits generalizations. 

Finally, and after adjusting for age and sex, psychomotor development tends to explains 58% of pre-school academic performance. It is interesting to notice the lower score when the analysis involves children in the formal education system. These findings reinforce the idea of the conceptual model of the psychomotor system [24,25,26], revealing higher scores in foundational psychomotor domains (*tonus*, *gross motor skills*, *manual praxis*). They demonstrate the progressive specialization of psychomotor skills, showing significant growth at early ages (3-6 years) and subsequent specialization in later ages. This dynamic underscores the need for a preventive psychomotor intervention plan in early childhood [9]. The adequate stimulation of psychomotor skills assumes a powerful and relevant influence not only in general development but also in (pre-)academic success. Body awareness and mastery allows for preparation for more adjusted responses to the increasing demands of complex and abstract knowledge. More active children will present more capabilities to be actively engaged in a more meaningful learning process, fostering the development of increasingly robust neural networks [2,4,7,63].

There is a strong relationship between psychomotor development and pre-academic skills compared to academic skills [1,2,3], maybe due to both periods’ characteristics and demands: during the pre-school period children experience relevant brain plasticity and sensitivity to experiences and environments, which facilitate the acquisition of new skills through lifelong learning [13,69]. Therefore, an early age full of high-quality learning experiences will tend towards a more successful academic trajectory [10,15,51,72]. This is aligned with Piaget and Kephart’s theories about the link between motor and cognitive development, emphasizing that the quality of higher-order thought processes is directly related to the quality of basic motor abilities [51]. 

Like any other study, several limitations should be considered in data interpretation. The reduced sample size demands some caution in the generalization, due to the impossibility of stratification and the geographic circumscription. Therefore, future research should replicate the study with representative and stratified samples and including children with and without specific learning needs, and compare low and high psychomotor and academic groups. This was a cross-sectional and correlational study and therefore causality cannot be inferred. The use of more specific instruments to analyze academic results (rather than SLSB) is another factor to be considered. This issue highlights the need to use narrower and more specific academic abilities for future research. Establishing the psychomotor profile of children within a scholastic context is urgent to understand how we can improve pedagogical methods to promote academic achievement [83] and contribute to the development of needed skills for the students of the 21st century, as well as the analysis of psychomotor interventions’ effectiveness in both psychomotor and academic performance during the life span. Analysis with other constructs (e.g., functional and cognitive–executive level) is another recommendation, as well as the identification of predictors of psychomotor development. Finally, it is suggested to identify and analyze moderators (family, motivation) of the relationship between psychomotor skills and learning achievements, and to deepen a model that could explain a major proportion of variation during the formal education period.

## 5. Conclusions

This study revealed a moderate correlation between psychomotor and academic domains, particularly at an early age, pointing to the interdependency between psychomotor development and learning and to indications that may predict academic success. Psychomotor skills appear to be relevant for learning, especially at an early age. This study’s findings impact early assessment and intervention. Using appropriate instruments to test the individual psychomotor level is relevant given the assumption that higher-order acquisitions are based on having learned and consolidate those that precede them. Having an individual psychomotor profile will complement medical and teachers’ reports, allowing for a focus on children-centered planning. A need to rethink and tailor-fit formal educational processes, instead of using cognitive and massified contents, emerges from our analysis. There is a need to continue to promote investment in early and complementary psychomotor and academic interventions embedded in a multidimensional model. Variables of sex, school year, and diagnosis follow the trends observed in previous studies, establishing a strong impact on the relationship between psychomotor development and school performance. Our findings corroborate the need for psychomotor intervention at pre-school age in a systematic and structured way, promoted by psychomotor specialists.

## Figures and Tables

**Table 1:** Correlations between NPmot.pt domains, PRE domains and SLSB domains.

	NPmot.pt Domains									
PRE Domains	T	GMS	L	MP	TG	HEC	SO	R	AA	NPmot.pt Total
Verbal	0.76 **	0.49 **	0.75 **	0.76 **	0.63 **	0.59 **	0.27 **	0.82 **	0.51 **	0.84 **
Quantitative concepts	0.79 **	0.63 **	0.80 **	0.77 **	0.44 **	0.47 **	0.12	0.59 **	0.50 **	0.80 **
Auditory memory	0.15	0.29 **	0.16	0.01	−0.39 **	−0.11	−0.33 **	−0.47 **	−0.12	−0.05
Visual perception–Constancy of form	0.52 **	0.48 **	0.58 **	0.52 **	0.49 **	0.36 **	0.09	0.38 **	0.48 **	0.56 **
Positions in space	0.16	0.36 **	0.26 **	0.12	−0.23 *	−0.01	−0.31 **	−0.29 **	0.10	0.05
Spatial Orientation	0.12	0.34 **	0.18	0.11	−0.17	−0.03	−0.31 **	−0.28 **	0.11	0.02
Visuomotor coordination	0.50 **	0.51 **	0.58 **	0.43 **	0.27 **	0.29 **	−0.15	0.22 *	0.30 **	0.45 **
Visual Perception Figure-Bottom	0.20 *	0.35 **	0.28 **	0.14	−0.15	0.02	−0.32 **	−0.28 **	0.07	0.07
PRE Total	0.45 **	0.54 **	0.52 **	0.40 **	0.06	0.20 *	−0.21 *	0.01	0.28 **	0.36 **
**SLSB domains**	T	GMS	L	MP	TG	HEC	SO	R	AA	NPmot.pt Total
Verbal Comprehension	0.13	0.04	0.24 **	0.13	−0.01	0.10	0.04	0.14 *	−0.04	0.14
Spatial Relations (Spatial Ability)	0.06	−0.01	0.18 *	0.04	−0.04	0.19 **	0.09	0.19 **	0.13	0.13
Quantitative Concepts (Numeric Ability)	0.12	−0.03	0.18 *	0.20 **	0.07	0.05	0.14 *	0.15 *	−0.09	0.15 *
Constancy of form (Perceptive Ability)	−0.01	−0.08	−0.02	0.03	0.01	0.03	0.04	0.06	0.11	0.03
Spatial Orientation (Perceptive Ability)	0.07	0.08	−0.11	0.02	−0.01	0.07	−0.09	−0.02	0.10	0.03
SLSB Total	0.13	0.01	0.17 *	0.14 *	0.00	0.15 *	0.07	0.18 *	0.08	0.17 *

** *p* ≤ 0.001; * *p* < 0.05 Legend: T—Tonus; GMS—Gross Motor Skills; L—Laterality; MP—Manual Praxis; TG—Tactile Gnosis; HEC—Hand-eye Coordination; SO—Spatial Orientation; R—Rhythm; AA—Auditory Attention.

**Table 2:** Pearson correlations between the NPmot.pt domains, PRE and SLSB, according to sex.

	NPmot.pt Domains																			
PRE Domains	T		GMS		L		MP		TG		HEC		SO		R		AA		Npmot.pt Total	
	M	F	M	F	M	F	M	F	M	F	M	F	M	F	M	F	M	F	M	F
Verbal	−0.32 **	−0.62 **	−0.74 **	−0.86 **	0.30 **	0.25 **	−0.80 **	−0.90 **	0.15 *	0.08	−0.39 **	−0.49 **	−0.52 **	−0.59 **	−0.15 *	−0.36 **	−0.51 **	−0.61 **	−0.62 **	−0.83 **
Quantitative concepts	−0.33 **	−0.60 **	−0.73 **	−0.84 **	0.30 **	0.25 **	−0.80 **	−0.89 **	0.13	0.08	−0.40 **	−0.50 **	−0.53 **	−0.61 **	−0.16 *	−0.39 **	−0.51 **	−0.61 **	−0.62 **	−0.83 **
Auditory memory	−0.16	0.06	−0.12	−0.06	0.06	0.01	−0.23 **	−0.22 **	−0.18 *	−0.19 **	−0.18 *	−0.19 **	−0.31 **	−0.34 **	−0.29 **	−0.41 **	−0.22 **	−0.18 *	−0.25 **	−0.24 **
Visual perception–Constancy of form	−0.32 **	−0.59 **	−0.71 **	−0.83 **	0.29 **	0.27 **	−0.78 **	−0.88 **	0.15	0.10	−0.39 **	−0.49 **	−0.53 **	−0.59 **	−0.17 *	−0.36 **	−0.50 **	−0.58 **	−0.61 **	−0.81 **
Positions in space	−0.25 **	−0.25 **	−0.43 **	−0.44 **	0.20 **	0.14	−0.56 **	−0.56 **	−0.03	−0.06	−0.30 **	−0.37 **	−0.52 **	−0.51 **	−0.27 **	−0.41 **	−0.35 **	−0.38 **	−0.48 **	−0.54 **
Spatial Orientation	−0.09	0.04	−0.08	−0.02	0.06	0.07	−0.20 **	−0.15	−0.08	−0.06	0.14	−0.16 *	−0.33 **	−0.28 **	−0.23 **	−0.24 **	−0.10	−0.08	−0.21 **	−0.16 *
Visuomotor coordination	−0.33 **	−0.57 **	−0.72 **	−0.80 **	0.30 **	0.24 **	−0.81 **	−0.84 **	0.13	0.07	−0.41 **	−0.48 **	−0.56 **	−0.61 **	−0.19 *	−0.38 **	−0.52 **	−0.58 **	−0.64 **	−0.81 **
Visual Perception Figure-Bottom	−0.28 **	−0.29 **	−0.55 **	−0.49 **	0.23 **	0.21 **	−0.67 **	−0.59 **	0.01	−0.01	−0.36 **	−0.37 **	−0.57 **	−0.54 **	−0.28 **	−0.41 **	−0.44 **	−0.40 **	−0.56 **	−0.57 **
PRE Total	−0.29 **	−0.36 **	−0.57 **	−0.56 **	0.25 **	0.20 **	−0.70 **	−0.67 **	0.04	−0.00	−0.36 **	−0.41 **	−0.57 **	−0.58 **	−0.26 **	−0.41 **	−0.44 **	−0.45 **	−0.57 **	−0.64 **
**SLSB domains**	**M**	**F**	**M**	**F**	**M**	**F**	**M**	**F**	**M**	**F**	**M**	**F**	**M**	**F**	**M**	**F**	**M**	**F**	**M**	**F**
Verbal Comprehension	0.46 **	0.61 **	0.79 **	0.85 **	−0.18 *	−0.23 **	0.86 **	0.90 **	−0.08	−0.07	0.46 **	0.51 **	0.55 **	0.61 **	0.25 **	0.41 **	0.55 **	0.62 **	0.72 **	0.84 **
Spatial Relations (Spatial Ability)	0.46 **	0.58 **	0.78 **	0.83 **	−0.15 *	−0.24 **	0.85 **	0.87 **	−0.08	−0.09	0.49 **	0.51 **	0.55 **	0.60 **	0.29 **	0.38 **	0.58 **	0.61 **	0.72 **	0.82 **
Quantitative Concepts (Numeric Ability)	0.45 **	0.62 **	0.79 **	0.85 **	−0.19 **	−0.23 **	0.87 **	0.90 **	−0.07	−0.08	0.45 **	0.51 **	0.56 **	0.61 **	0.25 **	0.41 **	0.55 **	0.62 **	0.72 **	0.85 **
Constancy of form (Perceptive Ability)	0.45 **	0.60 **	0.79 **	0.85 **	−0.20 **	−0.26 **	0.86 **	0.90 **	−0.08	−0.08	0.45 **	0.51 **	0.55 **	0.61 **	0.24 **	0.39 **	0.56 **	0.63 **	0.71 **	0.84 **
Spatial Orientation (Perceptive Ability)	0.45 **	0.62 **	0.79 **	0.86 **	−0.21 **	−0.26 **	0.86 **	0.90 **	−0.09	−0.07	0.45 **	0.52 **	0.55 **	0.60 **	0.23 **	0.40 **	0.55 **	0.64 **	0.71 **	0.85 **
SLSB Total	0.46 **	0.61 **	0.79 **	0.85 **	−0.19 *	−0.24 **	0.87 **	0.90 **	−0.08	−0.08	0.46 **	0.51 **	0.56 **	0.61 **	0.25 **	0.40 **	0.56 **	0.63 **	0.72 **	0.85 **

** *p* ≤ 0.001; * *p* < 0.05; Legend: M—Male; F—Female; T—Tonus; GMS—Gross Motor Skills; L—Laterality; MP—Manual Praxis; TG—Tactile Gnosis; HEC—Hand-eye Coordination; SO—Spatial Orientation; R—Rhythm; AA—Auditory Attention.

**Table 3:** Pearson correlations between the NPmot.pt domains, PRE and SLSB, according to year of schooling.

	NPMOT.pt Domains																														
Pre-School Diagnostic Tasks Domains	Tonus			Gross Motor Skills			Laterality			Manual Praxis			Tactile Gnosis				Hand-Eye Coordination			Spatial Orientation			Rhythm			Auditory Attention			Npmot.pt Total		
	1	2		1	2		1	2		1	2		1		2		1	2		1		2	1	2		1	2		1	2	
Verbal	−0.06	0.94 **		−0.39 **	0.69 **		−0.03	0.98 **		0.26 *	0.94 **		0.26 *	0.80 **			0.75 **	0.57 **		0.63 **	0.20		0.69 **	0.91 **		0.48 **	0.56 **		0.53 **	0.94 **	
Quantitative concepts	0.09	0.88 **		−0.03	0.73 **		0.17	0.87 **		0.33 **	0.87 **		0.20	0.52 **			0.21	0.56 **		0.43 **	0.03		0.48 **	0.65 **		0.44 **	0.59 **		0.47 **	0.82 **	
Auditory memory	0.14	0.17		0.44 **	0.30 *		−0.31 **	0.03		−0.29 *	0.14		−0.52 **	−0.34 **			−0.71 **	0.15		−0.74 **	−0.31 **		−0.69 **	−0.30 *		−0.43 **	0.28 *		−0.53 **	0.04	
Visual perception-Constancy of form	0.01	0.78 **		0.04	0.72 **		0.34 **	0.76 **		0.12	0.78 **		0.62 **	0.44 **			0.26 *	0.47 **		0.29 *	−0.05		0.19	0.55 **		0.36 **	0.59 **		0.29 *	0.71 **	
Positions in space	0.06	0.19		0.39 **	0.40 **		−0.18	0.13		−0.29 *	0.28 *		−0.18	−0.29 *			−0.44 **	0.15		−0.59 **	−0.35 **		−0.49 **	−0.18		−0.14	0.34 **		−0.37 **	0.10	
Spatial Orientation	0.17	0.11		0.43 **	0.35 **		0.34	0.03		−0.14	0.20		−0.03	−0.27 *			−0.31 **	0.05		−0.58 **	−0.36 **		−0.43 **	−0.21		−0.02	0.22		−0.21	0.03	
Visuomotor coordination	−0.04	0.76 **		0.27 *	0.71 **		−0.08	0.72 **		−0.14	0.76 **		0.04	0.41 **			−0.03	0.45 **		−0.43 **	−0.08		−0.11	0.51 **		0.12	0.56 **		−0.10	0.69 **	
Visual Perception-Figure-Bottom	0.02	0.27 *		0.37 **	0.40 **		0.17	0.17		−0.27 *	0.33 **		−0.04	−0.26 *			−0.42 **	0.19		−0.72 **	−0.31 **		−0.46 **	−0.15		−0.15	0.33 **		−0.37 **	0.16	
Pre-school Diagnostic Tasks Total	0.07	0.28 *		0.41 **	0.45 **		−0.02	0.20		−0.24 *	0.35 **		−0.07	−0.20			−0.36 **	0.18		−0.65 **	−0.32 **		−0.44 **	−0.09		−0.07	0.35 **		−0.31 **	0.18	
**BAPAE domains**	3	4	5	3	4	5	3	4	5	3	4	5	3	4		5	3	4	5	3	4	5	3	4	5	3	4	5	3	4	5
Verbal Comprehension	0.13	−0.31	0.43	0.04	−0.23	0.61	0.18 *	0.78 **	-	0.12	−0.12	0.48	−0.02	-		0.43	0.06	0.22	0.20	0.01	0.57	−0.09	0.13	−0.02	0.29	−0.04	0.12	−0.43	0.12	0.73	0.46
Spatial Relations (Spatial Ability)	0.04	-	-	−0.01	-	-	0.16 *	-	-	0.01	-	-	−0.05	-		-	0.16 *	-	-	0.06	-	-	0.17 *	-	-	0.10	-	-	0.10	-	-
Quantitative Concepts (Numeric Ability)	0.10	−0.31	0.58	−0.03	−0.39	0.20	0.16 *	0.52	-	0.19 *	−0.26	0.10	0.06	-		0.25	0.01	−0.11	−0.12	0.12	0.25	0.05	0.13	0.52	−0.25	−0.11	−0.15	−0.25	0.13	−0.23	0.23
Constancy of form (Perceptive Ability)	−0.04	−0.19	0.49	−0.08	0.00	−0.24	−0.02	−0.28	-	0.00	0.28	−0.01	0.02	-		−0.25	0.01	−0.43	0.04	0.03	−0.27	0.30	0.05	−0.28	−0.44	0.07	0.43	0.58	0.00	0.07	0.15
Spatial Orientation (Perceptive Ability)	0.06	−0.01	0.00	0.07	−0.20	0.50	−0.14	−0.09	-	−0.01	0.45	0.67 *	−0.01	-		−0.11	0.03	0.17	0.89 **	−0.01	0.19	−0.49	−0.03	−0.09	−0.26	0.08	0.70 *	−0.11	0.01	−0.03	0.13
BAPAE Total	0.11	−0.20	0.57	0.00	−0.32	0.52	0.13	0.51	-	0.11	−0.12	0.63	0.00	-		0.09	0.10	0.02	0.60	0.04	0.41	−0.20	0.16 *	−0.06	−0.31	0.03	0.75 **	−0.09	0.13	0.05	0.40

** *p* ≤ 0.001; * *p* < 0.05; Legend: 1—Pre-School (48–60 months); 2—Pre-School (60–72 months); 3—1st grade; 4—2nd grade; 5—>3rd grade.

**Table 4:** Pearson correlations between the NPmot.pt domains, PRE and SLSB, according to diagnosis.

NPMOT.pt Domains																				
Pre−School Diagnostic Tasks Domains	Tonus		Gross Motor Skills		Laterality		Manual Praxis		Tactile Gnosis		Hand−Eye Coordination		Spatial Orientation		Rhythm		Auditory Attention		Npmot.pt Total	
	N	Y	N	Y	N	Y	N	Y	N	Y	N	Y	N	Y	N	Y	N	Y	N	Y
Verbal	−0.64 **	0.04	−0.84 **	−0.36	0.30 **	0.23	−0.90 **	−0.36	0.11 *	0.19	−0.46 **	−0.23	−0.59 **	0.26	−0.26 **	−0.02	−0.57 **	−0.24	−0.82 **	0.10
Quantitative concepts	−0.63 **	0.04	−0.82 **	−0.36	0.30 **	0.23	−0.89 **	−0.36	0.10	0.19	−0.46 **	−0.23	−0.61 **	0.26	−0.28 **	−0.02	−0.57 **	−0.24	−0.82 **	0.10
Auditory memory	−0.03	-	−0.09	-	0.04	-	−0.23 **	-	−0.18 **	-	−0.19 **	-	−0.32 **	-	−0.36 **	-	−0.19 **	-	−0.26 **	-
Visual perception−Constancy of form	−0.62 **	0.04	−0.81 **	−0.36	0.31 **	0.23	−0.88 **	−0.36	0.12 *	0.19	−0.45 **	−0.23	−0.60 **	0.26	−0.28 **	−0.02	−0.55 **	−0.24	−0.81 **	0.10
Positions in space	−0.37 **	-	−0.45 **	-	0.18 **	-	−0.59 **	-	−0.05	-	−0.34 **	-	−0.53 **	-	−0.36 **	-	−0.36 **	-	−0.58 **	-
Spatial Orientation	−0.05	0.04	−0.06	−0.36	0.07	0.23	−0.18 **	−0.36	−0.07	0.19	−0.14 *	−0.23	−0.30 **	0.26	−0.25 **	−0.02	−0.09	−0.24	−0.20 **	0.10
Visuomotor coordination	−0.62 **	0.04	−0.80 **	−0.36	0.29 **	0.23	−0.88 **	−0.36	0.10	0.19	−0.46 **	−0.23	−0.62 **	0.26	−0.30 **	−0.02	−0.56 **	−0.24	−0.82 **	0.10
Visual Perception−Figure−Bottom	−0.41 **	0.04	−0.54 **	−0.36	0.24 **	0.23	−0.66 **	−0.36	−0.01	0.19	−0.37 **	−0.23	−0.58 **	0.26	−0.36 **	−0.02	−0.42 **	−0.24	−0.64 **	0.10
Pre−school Diagnostic Tasks Total	−0.46 **	0.04	−0.59 **	−0.36	0.24 **	0.23	−0.72 **	−0.36	0.01	0.19	−0.40 **	−0.23	−0.59 **	0.26	−0.35 **	−0.02	−0.45 **	−0.24	−0.68 **	0.10
**BAPAE domains**	**N**	**Y**	**N**	**Y**	**N**	**Y**	**N**	**Y**	**N**	**Y**	**N**	**Y**	**N**	**Y**	**N**	**Y**	**N**	**Y**	**N**	**Y**
Verbal Comprehension	0.65 **	0.42	0.83 **	0.73 **	−0.28 **	0.27	0.90 **	0.74 **	−0.11	0.30	0.47 **	0.66 *	0.60 **	0.20	0.30 **	0.49 *	0.58 **	0.64 **	0.83 **	0.55 *
Spatial Relations (Spatial Ability)	0.63 **	0.42	0.81 **	0.75 **	−0.26 **	0.23	0.88 **	0.74 **	−0.11 *	0.30	0.49 **	0.64 **	0.60 **	0.59	0.31 **	0.49 *	0.59 **	0.61 **	0.83 **	0.55 *
Quantitative Concepts (Numeric Ability)	0.64 **	0.43	0.83 **	0.75 **	−0.28 **	0.24	0.91 **	0.74 **	−0.10	0.30	0.47 **	0.65 **	0.61 **	0.54	0.30 **	0.49 *	0.58 **	0.62 **	0.83 **	0.55 *
Constancy of form (Perceptive Ability)	0.63 **	0.43	0.83 **	0.75 **	−0.30 **	0.24	0.90 **	0.74 **	−0.11	0.30	0.47 **	0.63 **	0.61 **	0.58	0.29 **	0.48 *	0.59 **	0.61 **	0.83 **	0.55 *
Spatial Orientation (Perceptive Ability)	0.64 **	0.43	0.83 **	0.75 **	−0.31 **	0.24	0.90 **	0.74 **	−0.11	0.30	0.47 **	0.64 **	0.60 **	0.56	0.28 **	0.49 *	0.59 **	0.62 **	0.83 **	0.55 *
BAPAE Total	0.64 **	0.43	0.83 **	0.75 **	−0.29 **	0.25	0.90 **	0.74 **	−0.11	0.30	0.47 **	0.65 **	0.61 **	0.53	0.30 **	0.49 *	0.59 **	0.62 **	0.83 **	0.55 *

** *p* ≤ 0.001; * *p* < 0.05; Legend: N—No; Y—Yes.

**Table 5:** Regression analyses between NPmot.pt and PRE domains and between NPmot.pt and SLSB domains.

Npmot.pt Domains	PRE Domains		SLSB Domains	
	β (95% Confidence Interval)	*p*	β (95% Confidence Interval)	*p*
Tonus	0.67 (0.29, 1.04)	**<0.001**	0.18 (−0.13, 0.50)	0.245
Gross Motor Skills	1.04 (0.34, 1.75)	**0.004**	−0.17 (−0.47, 0.13)	0.262
Manual Praxis	0.26 (−0.55, 1.07)	0.531	−0.07 (−0.33, 0.19)	0.595
Tactile Gnosis	0.07 (−3.68, 3.8)	0.969	−0.01 (−0.82, 0.80)	0.980
Hand–eye coordination	−0.79 (−2.27, 0.69)	0.292	0.30 (−0.114, 0.705)	0.156
Spatial Orientation	−1.44 (−2.09, −0.79)	**<0.001**	−0.108 (−0.373, 0.157)	0.424
Rhythm	−1.59 (−2.51, −0.68)	**<0.001**	0.199 (−0.112, 0.510)	0.209
Auditory Attention	3.68 (1.99, 5.38)	**<0.001**	−0.373 (−0.972, 0.226)	0.221

β Standardized coefficients Beta, adjusted to age and sex.

## Data Availability

No new data were created or analyzed in this study. Data sharing is not applicable to this article.
